# Co-Occurrence of Regulated, Masked and Emerging Mycotoxins and Secondary Metabolites in Finished Feed and Maize—An Extensive Survey

**DOI:** 10.3390/toxins8120363

**Published:** 2016-12-06

**Authors:** Paula Kovalsky, Gregor Kos, Karin Nährer, Christina Schwab, Timothy Jenkins, Gerd Schatzmayr, Michael Sulyok, Rudolf Krska

**Affiliations:** 1 BIOMIN Research Center, Tulln 3430, Austria; paula.kovalsky@biomin.net (P.K.); karin.naehrer@biomin.net (K.N.); christina.schwab@biomin.net (C.S.); timothy.jenkins@biomin.net (T.J.); gerd.schatzmayr@biomin.net (G.S.); 2 Department of Atmospheric and Oceanic Sciences, McGill University, Montreal QC H3A 0B9, Canada; greg@meteo.mcgill.ca; 3 Department of Agrobiotechnology (IFA-Tulln), University of Natural Resources and Life Sciences, Vienna 1180, Austria; rudolf.krska@boku.ac.at

**Keywords:** mycotoxin, secondary metabolites, survey, global, masked mycotoxins, emerging mycotoxins, concentration data

## Abstract

Global trade of agricultural commodities (e.g., animal feed) requires monitoring for fungal toxins. Also, little is known about masked and emerging toxins and metabolites. 1926 samples from 52 countries were analysed for toxins and metabolites. Of 162 compounds detected, up to 68 metabolites were found in a single sample. A subset of 1113 finished feed, maize and maize silage samples containing 57 compounds from 2012 to 2015 from 44 countries was investigated using liquid chromatography and mass spectrometry. Deoxynivalenol (DON), zearalenone (ZEN) and fumonisins showed large increases of annual medians in Europe. Within a region, distinct trends were observed, suggesting importance of local meteorology and cultivars. In 2015, median DON concentrations increased to 1400 μg·kg${}^{-1}$ in Austria, but were stable in Germany at 350 μg·kg${}^{-1}$. In 2014, enniatins occurred at median concentrations of 250 μg·kg${}^{-1}$ in Europe, at levels similar to DON and ZEN. The latter were frequently correlated with DON-3-glucoside and ZEN-14-sulfate. Co-occurrence of regulated toxins was frequent with e.g., enniatins, and moniliformin. Correlation was observed between DON and DON-3-glucoside and with beauvericin. Results indicate that considerably more than 25% of agricultural commodities could be contaminated with mycotoxins as suggested by FAO, although this is at least partly due to the lower limits of detection in the current survey. Observed contamination percentages ranged from 7.1 to 79% for B trichothecenes and 88% for ZEN.

## 1. Introduction

The contamination of agricultural commodities with mycotoxins (secondary fungal metabolites) is of global concern, due to their toxicity and impacts on animal health [1,2]. The most important genera producing mycotoxins and fungal secondary metabolites are *Aspergillus*, *Fusarium*, and *Penicillium* [3]. Contamination with mycotoxins by fungal growth occurs in the field, during storage and transportation. A highly concentrated, localised “hot-spot” (i.e., a highly inhomogeneous distribution of a toxin) can spoil an entire batch [4].

**Figure 1:**
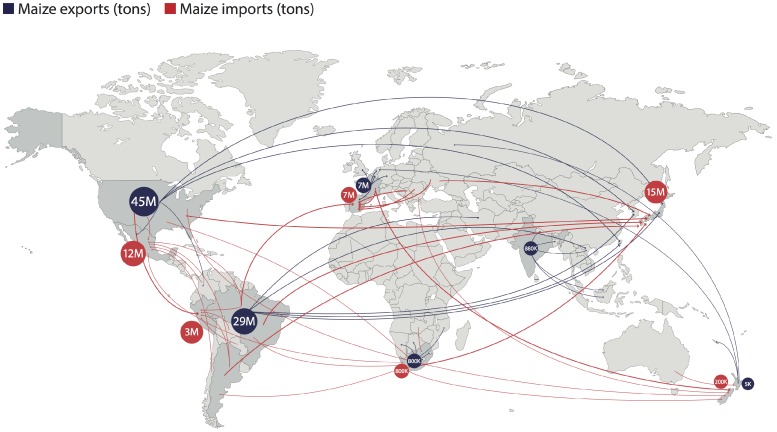
Simplified global maize trade for 2015. Map shows the largest importer (red) and exporter (blue) on each continent with the five largest countries of origin and destination, respectively [7].

The movement of animal feed products across the globe facilitated by international trade agreements requires constant monitoring of mycotoxin levels by authorities and traders; see Figure 1 for an example of the global maize trade. In 2014 compound feed production was 153 million tonnes in the European Union (EU–28), with global production having reached 964 million tonnes [5]. In 2014 compound feed accounted for 80% of all of the purchased feedstuffs in the EU [6].

### 1.1. Regulated Toxins and Mycotoxins with Guidance Levels

Legislation has been put in place for compounds commonly called “regulated toxins” and “mycotoxins with guidance levels”, which are comprised of aflatoxins (AFLA), some type A and B trichothecenes, zearalenone (ZEN), fumonisins (FUM) and ochratoxin A (OTA), because of their acute and chronic toxic effects. Values specified in feed are either maximum allowed levels or guidance values.

Animal consumption of commodities contaminated with AFLA causes extensive functional and structural damage to the liver, including links to liver cancer [8]. Livestock animals (e.g., poultry, swine) show sensitivity to AFLA [9]. While aflatoxin B${}_{1}$ is the most potent toxin and carcinogen, its congeners, nevertheless, also show high toxicity [10]. Among agricultural commodities, peanuts, maize and rice, mostly originating from subtropical and tropical regions are affected by elevated AFLA concentrations [11].

Trichothecenes are sesquiterpenes with an epoxy ring produced mainly, but not exclusively, by *Fusarium* species. Depending on the chemical structure more toxic, but less prevalent type A trichothecenes (e.g., T-2 and HT-2 toxins) and widely occurring type B trichothecenes (e.g., DON, nivalenol) are defined. Contamination typically occurs before harvest in maize and cereals. Toxicity mostly affects the gastrointestinal tract causing vomiting, diarrhea due to inhibition of protein synthesis [12]. Lower feed conversion ratios [13] and reduced feed intake have also been reported, especially at low contamination and chronic dose conditions.

The type B trichothecene DON, the most prevalent trichothecene in temperate climates is a stable molecule, thus moving through feed production intact. Formation of DON is commonly observed with concentrations up to the lower mg·kg${}^{-1}$. The main symptoms are vomiting (hence its common name “vomitoxin”), feed refusal, skin damage and hemorrhage [14,15] notably in swine. For poultry, feed contaminated with <5 mg·kg${}^{-1}$ DON results in a decreased immune response and increase of infectious diseases [16].

The type A trichothecenes T-2 and HT-2 toxins occur less often and at lower concentrations in feed than e.g., DON, but are more toxic. Young pigs exposed to T-2 toxin showed decreases in red blood cell and leucocyte count [17]. Low-concentration dosage increased immunotoxic effects [18], decreased body weight gain and possible tissue damage in poultry [19]. For a comprehensive summary see Grenier and Oswald [20]. HT-2 toxin is the main metabolite of T-2 toxin and of similar toxicity. Data for the two mycotoxins are usually presented together [21].

Like DON, ZEN is also produced by *Fusarium* species and found in the same group of crops under cool and wet conditions. It is, together with some of its metabolites, known to be the cause of hyperestrogenism, causing breeding problems notably in swine and poultry. Concentrations in cereals were observed in the μg·kg${}^{-1}$ range.

FUM are a group of *Fusarium* produced toxins, frequently co-occurring with DON and ZEN. Symptoms observed with contaminated feed are low appetite and activity, as well as pulmonary oedema in pigs [22]. Due to their stability, FUM are readily detected in processed feed. In poultry, effects on broiler chicks showed morphological changes such as reduced villus height [23].

Produced by *Aspergillus* and *Penicillium* species, OTA is a mycotoxin commonly produced in storage facilities, as opposed to previously discussed toxins, originating mainly from the field. It has nephrotoxic effects in mammals, notably swine, and is suspected to be a carcinogen [24]. In animals, especially poultry, ochratoxicosis is characterised by poor weight gain and decreased egg production [25]. Observed concentrations are in the lower μg·kg${}^{-1}$ range. OTA is readily transferred into finished feed products [26].

In general, some of the drastic acute symptoms described above were due to ingestion of large toxin quantities, which were linked to the consumption of heavily mould-infested feed. However, continuous low concentration exposure is also of relevance. For AFLA, chronic effects include the development of hepatitis and jaundice [9]. In general, chronic toxicity has been less investigated as were synergistic and additive effects due to co-occurrence of multiple compounds [20]. Available data is still scarce [27,28].

### 1.2. Masked Mycotoxins

The study of “masked mycotoxins”, i.e., plant metabolites of mycotoxins, or following Rychlik et al. [29]’s systematic definition “biologically modified” mycotoxins, is especially challenging since the chemical modifications introduced by the plant’s metabolism potentially has effects on both, toxicity (which could be increased or decreased compared to the parent toxin molecule) and analytical detectability. For the latter, masked toxins are either bound to carbohydrates or proteins and, therefore, not extractable with existing protocols aimed at the extraction of the toxin, or they are not detectable using established chromatography routines; hence their name “masked” mycotoxins; see [30]. Furthermore, because of structural similarities, some masked compounds, which sometimes differ in toxicity, are co-detected with the toxin itself by e.g., immunoassays [31]. Because of these analytical challenges and subsequent lack of established methodologies for routine testing, data are still scarce [32,33]. Among the group of masked mycotoxins, ZEN-14-sulfate and DON-3-glucoside are most commonly observed in feed. Their toxicological properties are currently being investigated, including the conversion of DON-3-glucoside to DON and ZEN-14-sulfate to ZEN by microbiota of the intestinal tract in an effort to assess the risks with exposure to masked mycotoxins [34]. ZEN-14-sulfate is a natural *Fusarium* metabolite [35]. Since it is readily hydrolysed, ZEN is produced upon ingestion by animals, triggering an estrogenic response typical for ZEN in e.g., swine. In contrast, the glucosides resist hydrolysis and are, therefore, not active.

### 1.3. Emerging Toxins

“Emerging toxins” are a group of chemically diverse mycotoxins for which to date no regulations exist. Ongoing studies employing advanced LC–MS/MS (liquid chromatography tandem mass spectrometry) for structure elucidation provide a steady stream of insights about newly discovered metabolites as do plant breeding efforts adapting to a changing climate [36]. Risk assessment studies are currently underway in preparation for legislation, if deemed necessary [37]. Commonly mentioned in this group are aflatoxin precursors, ergot alkaloids, enniatins, beauvericin (BEA) and moniliformin (MON). For a detailed list of individual substances grouped by these terms, see Table 1. Jestoi [38] has published an extensive review regarding this diverse set of compounds, summarising available data, analytical methods and toxicity studies available.

### 1.4. Co-Occurrence

Serrano et al. [39] investigated samples from the Mediterranean region and found contamination with multiple toxins, notably nivalenol and BEA in a number of cereal products with contamination rates between 33% and 95%, highlighting the prevalence of multi-mycotoxin contamination. Similar observations were made during other comprehensive studies focusing on animal feed samples, with 38%–75% of samples being contaminated with more than one toxin [40,41]. Among masked toxins DON-3-glucoside has been reported to frequently co-occur with DON in commodities such as wheat, maize and barley and is also formed during processing [42]. It is formed by the plant following DON production by *Fusarium* fungi [43].

Among *Fusarium*–produced toxins, co-occurrence is frequently observed for compounds such as ZEN, DON and FUM and synergistic effects of *Fusarium* were reported in the past [44]. These included reduced weight gain in pigs (DON and fumonisin B1) [45] and adverse effects on broiler chicks [19]. Sensitivity to DON, even at low dosages, and overall toxicity is determined by co-occurring compounds, such as FUM, present in feed [46]. Synergistic effects at concentrations close to EU guidance levels have been reported to impact antioxidant activity of cells [47]. Co-ocurrence of DON, ZEN and nivalenol was reported in cereal samples by Tanaka et al. [48]. Other, similar findings, on a global scale were summarised by Placinta et al. [49]. For ZEN and ZEN-14-sulfate, co-occurrence with DON and nivalenol is well established [50].

### 1.5. Regulations

In the EU, maximum allowable concentrations vary with commodity, degree of processing (e.g., maize, finished feed) and intended consumers (e.g., animal feed). Lowest levels are in place for AFLA with a maximum limit of 20 μg·kg${}^{-1}$, 5 μg·kg${}^{-1}$ for compound feed for dairy cattle and calves, sheep, piglets and young poultry animals [51]. In the EU, 1000 mg·kg${}^{-1}$ are the set maximum allowable level for ergot sclerotia. Recommended tolerance levels for ergot alkaloids in feed in Canada for swine are 4–6 mg·kg${}^{-1}$ and chicks at 6–9 mg·kg${}^{-1}$ [52].

For other compounds guidance levels have been put in place. FUM in animal feed has a set guidance level of 60 mg·kg${}^{-1}$, but with lower values for pigs (5 mg·kg${}^{-1}$) and poultry and young animals (20 mg·kg${}^{-1}$). Guidance values for DON (0.9–12 mg·kg${}^{-1}$), ZEN (0.1–2 mg·kg${}^{-1}$) and OTA (0.1–0.25 μg·kg${}^{-1}$) are significantly lower. The upper level provides a general value with exceptions applying for animals showing high sensitivity. Lowest values are typically established for swine, poultry and young animals [51]. Recommended values for the sum of T-2 and HT-2 toxins are lowest with 250 μg·kg${}^{-1}$ for compound feed, 500 μg·kg${}^{-1}$ for cereal products and 2000 μg·kg${}^{-1}$ for oat milling products [53].

### 1.6. Global Surveys

The analysis of global mycotoxin occurrence data is of particular interest, because it helps with the identification of geographical areas, which are highly contaminated and, thus, might affect global trade of agricultural commodities. It also supports the study of emerging toxins due to changing climatic conditions or specific meteorological events, such as exceptionally dry or wet growing seasons [54,55].

Previous survey reports focused mostly on regulated toxins and toxins with guidance levels and their co-occurrence among regulated mycotoxins, e.g., Streit et al. [40], Tanaka et al. [48], Murugesan et al. [56], Streit et al. [57], Rodrigues and Naehrer [58]. Sharman et al. [59] monitored MON concentrations on a global scale. While a limited number of global survey papers exist, other reports cover smaller geographic regions, e.g., Romania, Argentina and The Netherlands [48,60,61]. While these studies contribute to the availability of global data, different analytical methodologies employed make a comparison of concentrations challenging.

Recently increased awareness has led to the study of masked and emerging toxins. The need to investigate co-occurrence and possible correlation of regulated toxins, toxins with guidance levels and masked and emerging toxin concentrations has been stressed by Schatzmayr and Streit [62], Streit et al. [41] and Jestoi [38]. A summary of global survey data published during the past 5 years is provided in Table 2. During the last 5 years only a small number of true global surveys was carried out. Overwhelmingly, the focus of the reported work was on regulated toxins and toxins with guidance levels, using a variety of analytical methods, including relatively simple thin layer chromatography. This complicates direct comparisons due to differing limits of detection and analytical performance parameters, leading to potential underreporting due to different limits of detection (LOD). Van Der Fels-Klerx et al. [63] explicitly acknowledged this issue, and as a result the LOD for data coming from different sources in their study (i.e., national monitoring programmes) was fixed at the highest LOD most frequently reported for a specific toxin.

The resulting requirements call for an analytical methodology that is able to provide consistent high-quality and comparable results for a wide range of metabolites as co-occurrence and correlation of compounds are increasingly studied. The lack of information on masked and emerging toxins, including co-occurrence with regulated toxins and toxins with guidance levels calls for expanded survey data. In this way, new trends and emerging research questions that go hand-in-hand with increasing frequency of extreme weather events and a changing climate can be efficiently addressed [54,55,76].

This study presents global survey data for the years 2012–2015 for regulated toxins, toxins with guidance levels and selected masked and emerging toxins and metabolites. Toxin concentrations were summed up creating groups of toxins and metabolites that were studied as a sum (e.g., AFLA were reported as the sum of concentrations of aflatoxins B1, B2, G1 and G2; for details regarding the other groups, see Table 1). Concentrations were determined with a single liquid chromatography mass spectrometric method. Mycotoxin and metabolite concentrations of 57 compounds in finished feed, maize and maize silage were investigated in 1113 samples from 46 countries. Representative results from regions and countries across the globe were compiled. Specifically, the co-occurrence and correlation of regulated mycotoxins and toxins with guidance levels with selected masked and emerging toxins and metabolites was investigated.

## 2. Results

Results of regulated toxins, toxins with guidance levels, masked and emerging toxin concentrations from 1113 samples of global origin from 3 commodities (finished feed, maize and maize silage) collected from 2012 to 2015 are presented. Table 1 and Table 5 provide a description and basic statistical information of the data set and the subsets investigated. Samples contained on average 16 and up to 35 out of 57 compounds at the same time. The number of metabolites found in a single sample was 35 in finished feed, 29 in maize and 28 in maize silage. The lower part of Table 5 provides subset information with regard to number of samples available per matrix.

Regarding the reported trends for the years which observations were reported for, it has to be noted that because the data are limited to only four consecutive years and it is well known that especially for mycotoxin contamination there are large year–to–year variations, no long–term trends can be deduced at this point.

Results from subsets with sample numbers greater than 40 were reported in detail; for details regarding the number of samples in each subset, see Table 5. If, for completeness, subsets with lower sample number were chosen, this was clearly indicated. Summary data in Table 1 highlights global relevance of ZEN and DON in the investigated matrices with 88% and 79% of 1113 samples contaminated with concentrations above the threshold. Prevalence of the related masked toxins DON-3-glucoside (70%) and ZEN-14-sulfate (47%) was high. Among emerging toxins, MON and BEA showed high occurrence in 79 and 83% of samples, respectively. Slightly lower occurrence was observed for enniatins with enniatin A, A1, B and B1 occurring in 32%, 59%, 71% and 69% of samples.

**Figure 2:**
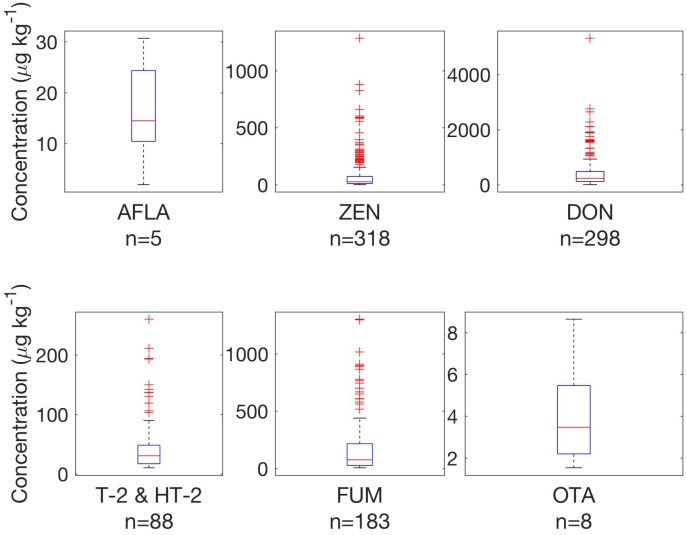
Survey results for regulated toxins and toxins with guidance levels in 335 finished feed samples in Central Europe above defined thresholds listed in Table 1. *n* provides number of samples. Boxplots follow definition by McGill et al. [77].

Detailed regional results were most useful to study trends and concentrations for the purpose of regulation and monitoring. Figure 2 provides a summary of concentrations of regulated toxins and toxins with guidance levels in Central Europe. Maximum observed concentrations from Table 1 and data available from Figure 2 show that observed concentrations are relevant for regulatory purposes and animal health.

**Figure 3:**
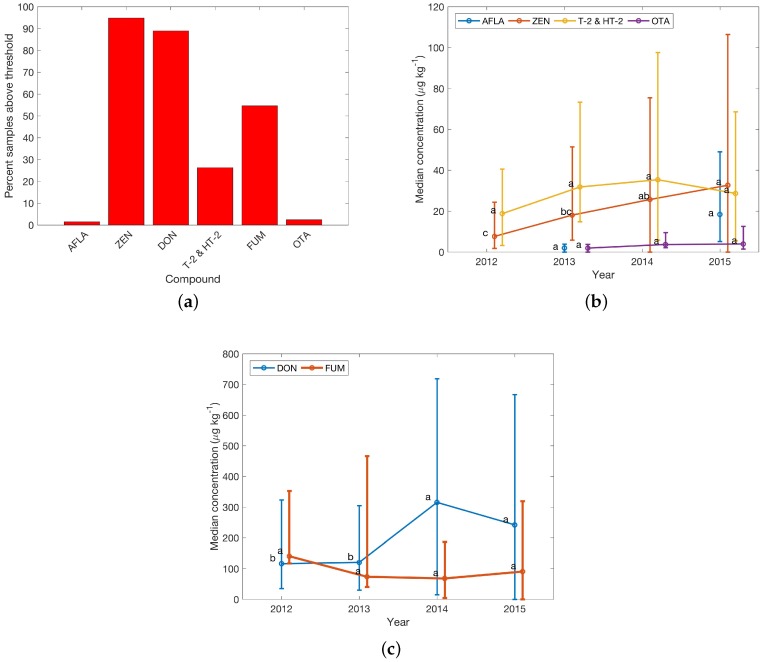
Survey results for regulated toxins in finished feed samples in Central Europe. (**a**) Percentage of samples with concentrations above thresholds; see Table 1 for details; (**b**) and (**c**) Yearly median concentrations from 2012 to 2015 (missing point indicates that no data were available). Error bars reflect the Wilcoxon confidence interval (CI). Lower error were replaced with the median, if the Wilcoxon CI would have resulted in negative concentrations. Significance codes show differences between yearly medians from a Kruskal–Wallis test result. Different letters indicate a significant difference between the groups. Data points were offset on the *x*-axis for clarity. Sample numbers for calculation of the median of each year are availale in Table 3.

This occurrence pattern is further elaborated on in Figure 3. The percentage of samples with concentrations above the threshold was low (<5%) for AFLA and OTA, but the percentage of above-threshold concentrations in samples for the other four regulated mycotoxins and mycotoxins with guidance levels was greater than 50%, and high for DON and ZEN (with >80% of investigated finished feed samples showing concentrations above threshold (see Figure 3a). Regarding yearly median concentrations 2014 and 2015 showed marked increases for DON and ZEN and FUM with doubled median concentrations compared to 2012 and 2013 (Figure 3b,c). Significance codes indicate significant differences between yearly medians as a result of Kruskal–Wallis tests, while error bars provide Wilcoxon confidence intervals. Different letters indicate a significant difference between the groups. The size is due to a large concentration range and few available samples (e.g., for 2012). Concentrations for other regulated toxins and toxins with guidance levels remained stable.

**Figure 4:**
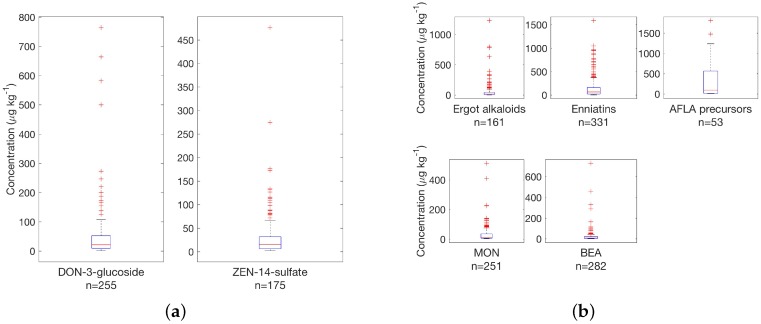
Survey results for (**a**) masked and (**b**) emerging toxins in finished feed samples from Central Europe (335 samples) above threshold concentrations; see Table 1 for details.

The situation regarding masked and emerging toxins and secondary metabolites is illustrated in Figure 4. Compared to regulated toxins and toxins with guidance levels, masked toxin concentrations were in a range of up to 800 μg·kg${}^{-1}$ with a larger number of samples at concentrations around <300 μg·kg${}^{-1}$ in finished feed samples.

**Figure 5:**
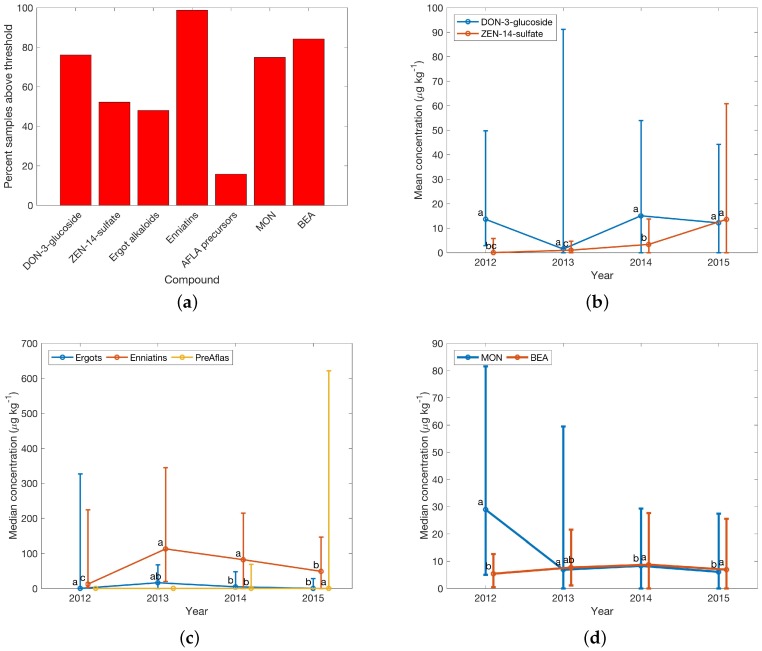
(**a**) Survey results for masked (DON-3-glucoside and ZEN-14-sulfate) and emerging toxins in finished feed samples from Central Europe above threshold levels; see Table 1 for details. Subfigures (**b**) masked, (**c**) and (**d**) show yearly median data for emerging toxins for the years 2012–2015 in Central Europe. Error bars reflect the Wilcoxon confidence interval (CI). Lower error were replaced with the median, if the Wilcoxon CI would have resulted in negative concentrations. Significance codes show differences between yearly medians from a Kruskal–Wallis test result. Different letters indicate a significant difference between the groups. Data points were offset on the *x*-axis for clarity. Sample numbers for calculation of the median of each year are available in Table 3.

Results for masked and emerging toxins are complemented in a similar fashion as shown in Figure 3 to highlight the percentage of samples in finished feed above the defined threshold; see Figure 5a. Enniatins were ubiquitous in finished feed samples and MON and BEA were found in more than 70% and 80% of samples, respectively, i.e., very similar to DON and ZEN regarding occurrence, underlining the importance to study emerging toxins and secondary metabolites.

**Figure 6:**
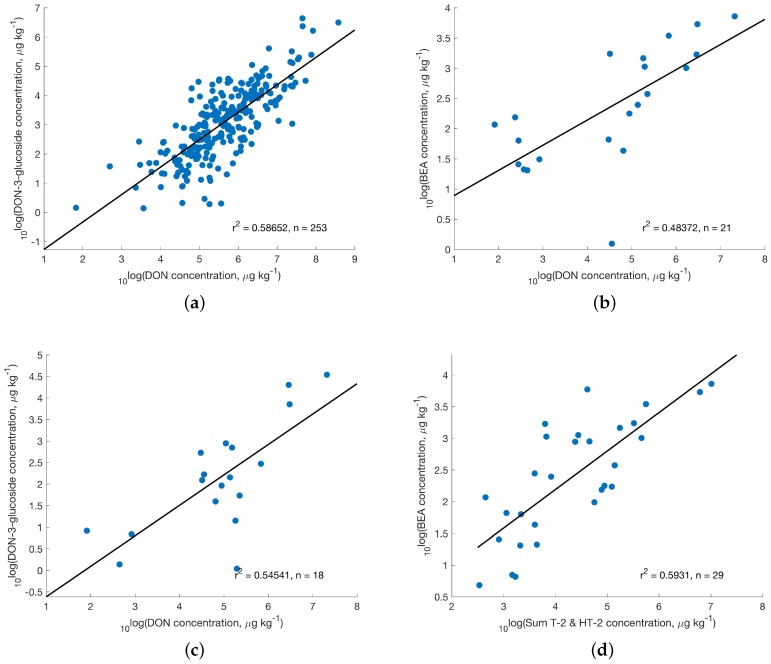
Correlation of (**a**) DON and DON-3-glucoside in Central Europe; (**b**) DON with BEA (Eastern Europe); (**c**) DON with DON-3-glucoside (Eastern Europe) and (**d**) sum of T-2 and HT-2 toxins with BEA in finished feed samples (Eastern Europe).

Correlation analysis was conducted to investigate how concentrations varied between compounds and if correlation was observed. In accordance with Van Der Fels-Klerx et al. [63] a correlation coefficient >0.5 was considered high enough to be reported. In finished feed samples from Central Europe a correlation coefficient of 0.6 between DON and DON-3-glucoside was observed (Figure 6a). For clarity, both axes were plotted in logarithmic form. Additional correlation data of regulated toxins with emerging compounds in Eastern Europe are found in Figure 6.

**Figure 7:**
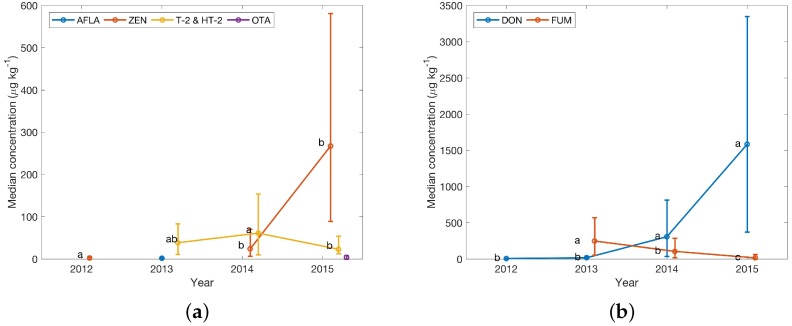

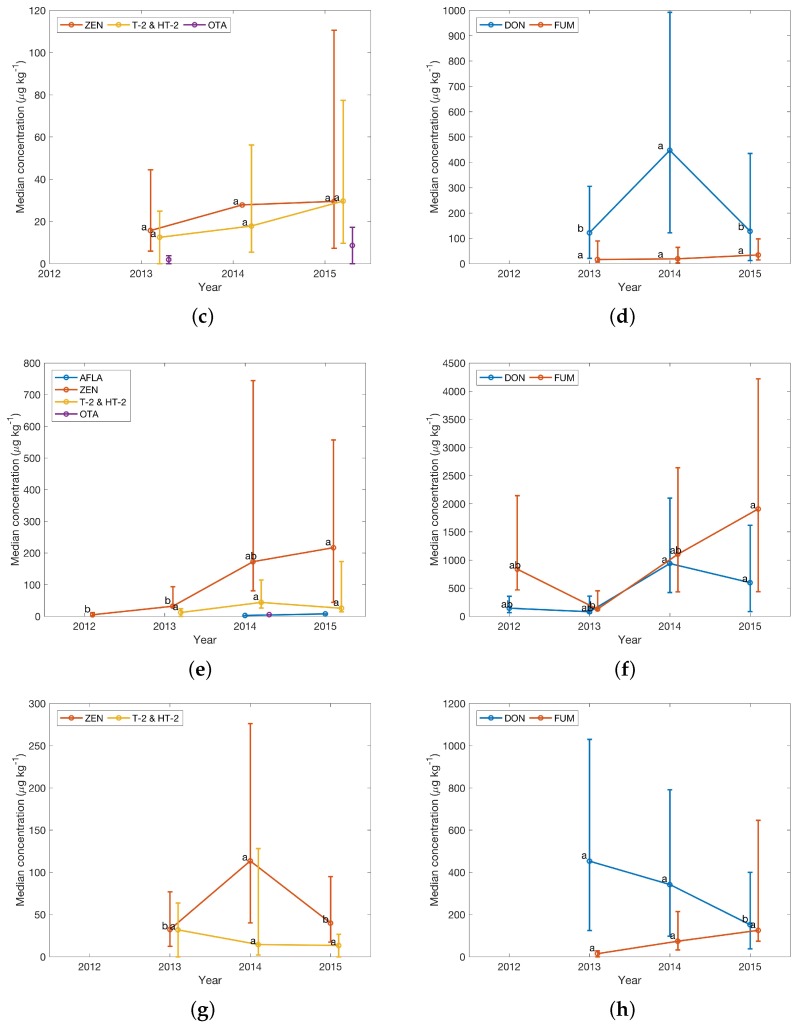
Yearly median concentrations of regulated toxins and compounds with guidance levels in finished feed from (**a**) and (**b**) Austria, (**c**) and (**d**) Germany from 2012 to 2015. Error bars reflect the Wilcoxon confidence interval (CI). Lower error were replaced with the median, if the Wilcoxon CI would have resulted in negative concentrations. Significance codes show differences between yearly medians from a Kruskal–Wallis test result. Different letters indicate a significant difference between the groups. Data points were offset on the *x*-axis for clarity. Sample numbers for calculation of the median of each year are availale in Table 3. Yearly median concentrations of regulated toxins and compounds with guidance levels in finished feed from (**e**) and (**f**) Italy, and (**g**) and (**h**) The Netherlands from 2012 to 2015. Error bars reflect the Wilcoxon confidence interval (CI). Lower error were replaced with the median, if the Wilcoxon CI would have resulted in negative concentrations. Significance codes show differences between yearly medians from a Kruskal–Wallis test result. Different letters indicate a significant difference between the groups. Data points were offset on the *x*-axis for clarity. Sample numbers for calculation of the median of each year are available in Table 3.

Country-specific data provide information about differences within a geographic region as demonstrated in Figure 7. The subplots show clear differences in observed yearly medians, e.g., for DON in Austria and Germany (1500 vs. 400 μg·kg${}^{-1}$ in 2014). Notable are also differences between Italy and Austria regarding yearly median FUM concentrations (1500 vs. <100 μg·kg${}^{-1}$) in 2015.

## 3. Discussion

### 3.1. Finished Feed in Central Europe

Box plots in Figure 2 illustrate occurrence and concentrations of regulated toxins and toxins with guidance levels in samples from Central Europe [77]. A large number of samples showed detectable concentrations for all compounds, although the majority of samples showed low concentrations, especially when compared to the maximum levels and guidance values. While overall the number of infected samples, i.e., with concentrations above the detection limit is quite high, this is comparable with other recent studies, e.g., reports by Streit et al. [57] for feed samples and Van Asselt et al. [67] for maize samples, where authors have chosen similar reporting limits for LC–MS/MS data, thus making comparisons feasible. The report by Van Asselt et al. [67] also revealed a high percentage of contaminated samples (e.g., >80% samples were contaminated with DON in 2006 and 2007. Information provided in this study (e.g., in Table 1) shows additional detail on concentration distribution, similar to data by Van Der Fels-Klerx et al. [63] for selected type A and B trichothecenes in cereal samples, restricted to Northern Europe. Figure 2 highlights that DON and ZEN were found at above-threshold concentrations in finished feed. Samples contained a sum of T-2 and HT-2 toxin concentrations above-threshold (up to 250 μg·kg${}^{-1}$). FUM with was found at maximum concentrations of 1200 μg·kg${}^{-1}$. Overall, legal limits and guidance values for regulated toxins and toxins with guidance levels are not reached for the vast majority of samples. Single samples exceed regulations (e.g., 5 samples with AFLA concentrations between 10 and 30 μg·kg${}^{-1}$), but given the overall samples analysed for Central Europe (*n* = 335) the percentage (1.5%) remains low. For DON, 27 samples (8.1%) showed concentrations above 0.9 ppm, the lowest guidance level established for piglets and calves. For all other regulated mycotoxins the established guideline values were not exceeded. The general occurrence and level of contamination with very high concentrations for few samples confirms data from previous studies [67]. Care has to be taken for reports stating the percentage of contaminated samples to ensure that comparable lower reporting limits for positive results were chosen, but even in older studies high percentages of positives were reported, e.g., 58% for ZEN in corn from a survey conducted in 19 countries [78].

As indicated in Table 4 there is a high degree of co-occurrence for both investigated masked toxins of samples containing both compounds. Typically, between 50% and (in some cases) almost all samples contained 3 or more (out of 6) regulated toxins or toxins with guidance levels, both masked toxins and 3 or more (out of 5) emerging toxins. This is found for all matrices investigated, where enough samples were available for a detailed assessment. Concentrations above 1 μg·kg${}^{-1}$ were observed in 30%–40% of finished feed samples. A previous report investigating co-occurrence of DON and the masked DON-3-glucoside [79] in samples from Central European countries also found a very high degree of co-occurrence with all 77 field samples containing both compounds.

With the exception of AFLA precursors and ergot alkaloids, all other investigated masked and emerging compounds occur in between 79% and 98% of samples, thus showing a high degree of co-occurrence with the most prevalent regulated toxins and toxins with guidance levels (i.e., DON, ZEN and FUM). Overall, a high degree of contamination was observed, which is due to low detection limits, but in line with previous studies on smaller sample sets, e.g., for enniatins among the emerging compounds and toxins with guidance levels, e.g., DON [41]. Maximum concentrations were in the 500–1500 μg·kg${}^{-1}$ range for 25 (7.6%) samples containing a summarised enniatin concentration >500 μg·kg${}^{-1}$. Yearly medians were in the 30 and 100 μg·kg${}^{-1}$ range for masked and emerging compounds, respectively and quite variable between 2012 and 2015. The high occurrence of masked and emerging compounds in finished feed samples from Central Europe is further illustrated in Figure 5a. With the exception of AFLA precursors, close to 50% or more samples showed concentrations >*t* for all investigated compounds. European samples containing AFLA, either the aflatoxins themselves or the precursors, were generally rare showing the lowest percentage of positives of all regulated toxins or toxins with guidance levels, which was comparable to previous reports Rodrigues et al. [64]. A fairly large number of samples also showed high to very high concentrations between 100 and 1500 μg·kg${}^{-1}$ for enniatins, ergot alkaloids and AFLA precursors. Occurrence of MON and BEA is similar with about two thirds of samples contaminated, but at lower concentrations up to 700 μg·kg${}^{-1}$.

The analysis for other regions and selected countries from Table 5 was conducted in an identical fashion; the following subsection provides a summary of results obtained from corresponding plots (data are shown in the supplementary information for the regions discussed).

### 3.2. Finished Feed in Global Regions and Countries

The situation for regulated toxins in other European regions was in general similar to that in Central Europe, with some notable differences. Contamination with DON and ZEN were still of concern (as these toxins are of global relevance), but was less prevalent in Eastern Europe at lower concentrations, i.e., less than 400 and up to 1500 μg·kg${}^{-1}$. Contamination with FUM was in a similar concentration range (up to 1500 μg·kg${}^{-1}$). A similar situation was also observed for Southern Europe.

Generally, very few samples were contaminated with AFLA in Central and Eastern Europe. The maximum concentration found in a single sample was almost three times higher in Eastern compared to Central Europe with 90 and 30 μg·kg${}^{-1}$, respectively. Northern and Southern Europe showed markedly lower maximum concentrations at 3 and 7 μg·kg${}^{-1}$, respectively. However, for these single samples established limits for young animals such as piglets and calves were exceeded. Therefore, a continued monitoring of AFLA levels is advisable, especially as finished feed varies in composition [80].

Samples from Central and Southern Europe showed the maximum ZEN concentration >1000 μg·kg${}^{-1}$, especially samples originating from Germany, Austria and Italy, which indicates high prevalence in Central Europe. In a report by Streit et al. [40] ZEN in feed materials in Europe was found to be at maximum concentration of 1045 μg·kg${}^{-1}$, so very much comparable with the data presented here. A previous report summarising occurrence of ZEN in different matrices found similar concentrations, e.g., max. 950 μg·kg${}^{-1}$ in feeds and grains [28]. Samples from North America and Northern Europe, at 800 and 400 μg·kg${}^{-1}$, followed. Just like DON, almost all samples contained ZEN at concentration levels >*t*, regardless the region or country of origin, but generally not at levels exceeding regulations. The least contamination was observed for OTA with low occurrence (typically around 10%), but individual samples with concentrations of up to 3000 μg·kg${}^{-1}$. The sum of T-2 and HT-2 toxins was in the 100–200 μg·kg${}^{-1}$ range.

In other non-European regions, OTA concentrations were comparatively elevated such as in samples from the Middle East (*n* = 23) and Africa (*n* = 24), with the caveat that sample numbers were lower. In samples from South America, South Africa and the Middle East, FUM was more prevalent than DON with a larger percentage containing FUM than DON above the threshold, e.g., 95% of samples containing FUM and 80% containing DON for South America. These results are mirrored in a report, where 94% of 224 samples tested positive for FUM and only 13% of 130 samples contained DON [58]. Contamination of finished feed with AFLA in African samples was of concern with >35% of samples at concentrations >*t* and a maximum concentration of 60 μg·kg${}^{-1}$, even though the number of samples was low (*n* = 24). Therefore, special precautions are required, when importing feed from Africa to ensure that established limits are met [81]. Samples from South Africa were not included here, as only 5% of samples showed notable concentrations of AFLA with a much larger number of samples (*n* = 74).

Regarding masked and emerging toxins, samples from Eastern Europe showed similar concentrations compared to Central Europe for masked mycotoxins in finished feed. Most concentrations were <50 μg·kg${}^{-1}$ with maxima of 90 μg·kg${}^{-1}$ (DON-3-glucoside) and 250 μg·kg${}^{-1}$ (ZEN-14-sulfate). For emerging toxins, the situation was quite similar in Eastern Europe compared to Central Europe. Concentrations of enniatins (with exception of a single highly contaminated sample) were generally lower, at around 500 μg·kg${}^{-1}$ for the higher contaminated samples. In Northern Europe a single, highly contaminated sample of AFLA precursors at 12,000 μg·kg${}^{-1}$ was found, with 15 samples containing AFLA precursors. Occurrence of high levels of AFLA precursors without elevated AFLA concentrations indicated microbial producers other than aflatoxigenic *Aspergillus* species. Sterigmatocystin is produced by approximately 15 different fungal species the most important one being *A. versicolor* [82]. It was observed that MON and BEA occurred at higher concentrations and more often in samples from Northern Europe, reaching concentration levels similar to DON, thus making all three compounds relevant for e.g., monitoring [83]. Low concentrations of <150 μg·kg${}^{-1}$ were found for emerging toxins in South Africa with MON showing highest concentrations at about 100 μg·kg${}^{-1}$. The situation was similar in Southern Europe, where MON showed highest concentrations at <100 μg·kg${}^{-1}$.

The number of contaminated samples generally suggest a high degree of co-occurrence, e.g., 298 of 335 samples in Central Europe showed concentrations >*t* for DON and 318 for ZEN; see Figure 2 and Table 4. The situation in other global regions is quite similar (e.g., for enniatins, BEA and MON in South Africa with 51, 64 and 71, respectively out of 74 samples). However, correlation of concentrations between regulated toxins, toxins with guidance levels and emerging toxins was less often observed as Table 4 demonstrates. DON is usually well correlated with DON-3-glucoside and so is ZEN with ZEN-14-sulfate. No correlation for ZEN with ZEN-14-sulfate was observed in samples from South Africa. Regarding AFLA precursors and AFLA, however, generally no correlation was observed, except for African finished feed data (for a relatively low sample number of *n* = 24) and maize samples in South Africa and South America. A larger number of notable correlations with r${}^{2}$ around 0.5 was found in samples from Eastern Europe for DON and the sum of T-2 and HT-2 toxins with the masked compound DON-3-glucoside and the emerging toxin BEA; see Figure 6 and Table 4 for details, where also single cases of MON correlated with regulated toxins are listed (e.g., for DON and ZEN, respectively in maize from South America).

### 3.3. Yearly Median Concentrations 2012–2015

For finished feed samples yearly median concentrations show some marked changes between 2012 and 2015. Figure 4c shows marked increases for DON in Central Europe for the years 2014 and 2015, which are significantly different compared to 2012 and 2013. In Eastern Europe, the median yearly FUM concentration was high (up to 600 μg·kg${}^{-1}$) in 2013, followed by an increase in DON for 2014. Samples from Northern Europe also showed median yearly DON increases similar to Central Europe. FUM concentrations were higher than DON for Southern Europe. A notable contrast was seen for neighbouring Germany and Austria, where DON concentrations decreased in 2015 for the former, but strongly increased for the latter (see Figure 7) at much higher concentrations. Significant differences are also seen for Italy, neighbouring Austria and The Netherlands, neighbouring Germany. This suggests that regional climate and cultivars might play an important role for infection rates. Therefore, it is advisable to be careful, when pooling data ensuring that data subsets are fit for purpose, resulting in different subsets for e.g., regulatory or environmental impact studies.

Overall, DON and FUM were the dominating compounds on a regional as well as country scale. Other regulated toxins and toxins with guidance levels only played a secondary role regarding yearly medians and the overall toxin load. However, there were countries with low yearly medians, e.g., Russia for DON in the 50–100 μg·kg${}^{-1}$ range or South Africa for DON with 50–300 μg·kg${}^{-1}$ yearly median concentrations.

Yearly median concentrations of masked and emerging compounds were quite variable, lacking some of the distinctive trends, e.g., observed for Central Europe for the years 2014 and 2015, where large increases in DON and FUM were observed (see comparison of Figure 5b–d and Figure 7 further supporting the hypothesis that co-occurrence does not imply correlation. Median concentrations were usually by a factor of 10 (emerging toxins) and 100 (masked mycotoxins) lower than e.g., regulated compounds for the same observation period. Another notable observation was a 5-fold increase to an median 250 μg·kg${}^{-1}$ of enniatin concentrations in Eastern European samples.

A large source of variability could be in the types of finished feed sampled and variation in the composition of each of the feeds. However, the extent of signal suppression was not significantly higher e.g., in feed compared to the other matrices. It is mostly relative matrix effects that are typically a source of concern, but these were considered to be sufficiently low for the purpose of this manuscript. These concerns add to to the variability in the data, generally increasing the difficulty of achieving statistical significance and introducing some potential for unintended bias in results. The finished feed types include poultry, swine and ruminant feed. These compound feeds can indeed vary in their contents (between and within feed types). A major source of variability between years can be the number of samples per country which is a large potential source of unintended bias. This is the reason for showing the individual countries in Figure 7 for regulated toxins and mycotoxins with guidance levels. Overall, the chosen thresholds are realistic, as they reflect the concentration that may be reliably determined leaving a safety margin for variation of the instrument’s performance and the complexity of the sample.

Due to the already discussed year-to-year variations, long-term trends could not be identified. Furthermore, the country of origin for the raw ingredients of finished feed samples is not necessarily also the country of origin, which makes the evaluation of contamination levels for specific geographical regions challenging.

### 3.4. Maize and Maize Silage

The observations of finished feed concentrations for the years 2012–2015 for regulated, masked and emerging toxins set a general trend that is largely observed for maize and maize silage, though with some key differences.

For regulated toxins and toxins with guidance levels in maize and maize silage from Central Europe a dozen samples out of 78 showed very high concentrations of ZEN up to 10,000 μg·kg${}^{-1}$. Concentrations were lower in maize silage (up to 3000 μg·kg${}^{-1}$ for ZEN), but otherwise similar trends were observed. The situation was analogous for DON. High yearly medians of 2500 μg·kg${}^{-1}$ for DON in maize silage were observed in 2014, which was also the case for maize, but at a higher concentration level of 4000 μg·kg${}^{-1}$. Maize samples in South Africa showed regulated toxins and toxins with guidance levels in a similar concentration range as in Central Europe with lower maxima (e.g., 8000 μg·kg${}^{-1}$ for a single DON sample). It has been reported previously that in maize high concentrations of AFLA were observed [61]. The presented data show high concentrations for individual samples only, for both, AFLA and AFLA precursors for all investigated regions. Even in maize samples from South America, only 11 out of 77 samples contained concentrations >1 μg·kg${}^{-1}$ with 3 samples featuring very high concentrations around 200 (for 2 samples) and a single sample at 1300 μg·kg${}^{-1}$.

Low concentrations were observed (<150 μg·kg${}^{-1}$) for emerging toxins in maize in South Africa with MON at highest concentrations at about 400 μg·kg${}^{-1}$ for the 2012–2015 years. The situation was similar in Southern Europe, where MON showed highest concentrations between 2012 and 2015, but generally at <100 μg·kg${}^{-1}$. In both cases only very few samples showed these elevated concentrations. Generally, a higher incidence of MON and BEA was observed in South African maize, whereas the occurrence of AFLA precursors, enniatins and ergot alkaloids was generally low. Overall, concentrations of emerging toxins in South American maize samples were lowest.

For masked toxins, maize silage showed the lowest concentrations of the three matrices with a range of 300–600 μg·kg${}^{-1}$ for the highest concentrations. Otherwise observations are similar to maize. Maximum concentrations in South African maize were higher for few individual samples with 1000 and 300 μg·kg${}^{-1}$ for DON-3-glucoside and ZEN-14-sulfate, respectively. In South America, maximum concentrations were even higher with 900 and 1200 μg·kg${}^{-1}$ for DON-3-glucoside and ZEN-14-sulfate, although yearly medians were well <100 μg·kg${}^{-1}$ since only very few samples were affected. 2014 and 2015 showed medians of 30 μg·kg${}^{-1}$ with virtually no masked toxins detected in 2013.

### 3.5. Type A and B Trichothecenes

The overall occurrence of type A and B trichothecenes was assessed by summation of compounds as described in Table 1. Trichothecenes occur together in about 40% of samples, with concentrations of type B trichothecenes being higher (9 samples showing concentrations between 4500 and up to 16,000 μg·kg${}^{-1}$). Maximum concentrations for type A trichothecenes on the other hand were 30 times lower in the 500 μg·kg${}^{-1}$ range. 55% of samples were contaminated with type A trichothecene concentrations >*t* and 80% samples contained type B trichothecenes. Guidance values for the sum of trichothecenes currently do not exist, so a comparison was made with the guidance value of DON alone. The sum of type A and, the sum of type B trichothecenes exceeded the guidance value for DON in 100+ samples; thus a detailed investigation of the impact of multi-toxin contamination is advisable due to the high degree of co–occurrence. For DON alone, concentrations around 3500 μg·kg${}^{-1}$, a level, which has previously shown adverse effects in animal studies (e.g., see [16]) were observed in all matrices investigated, e.g., in maize samples from South Africa and South America.

**Figure 8:**
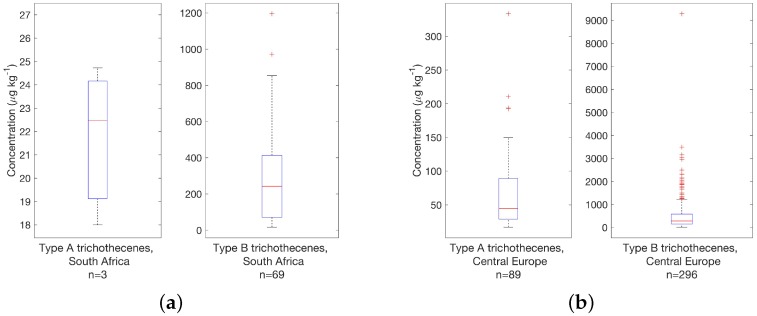
Survey results for type A and type B trichotheces from (**a**) South Africa and (**b**) Central Europe in finished feed samples above threshold concentrations; see Table 1 for details.

Type A and B trichothecenes were typically both found in most investigated finished feed samples with South Africa being a notable exception. Figure 8 compares occurrence (as sum of the respective trichothecene class indicated in Table 1. The absence of type A trichothecenes is clearly illustrated, since these were found in only in 3 samples, whereas type B trichothecenes are found in 69 out of 74 samples. While occurrence of T-2 toxin has been reported (e.g., by Placinta et al. [49], little quantitative data exists. T-2 and HT-2 toxins (and also DON) were determined in 92 commercial feed samples in a recent study by Njobeh et al. [72] and none of the samples contained these toxins, with the caveat that stated limits of detection were comparable with data reported here at 1 and 2.5 μg·kg${}^{-1}$, respectively. Overall data on A trichothecenes in South Africa remains scarce.

Trichothecene concentrations found in maize samples were similar to those finished feed. The situation for maize silage generally resembled maize with regard to concentration ranges and medians. Specifically, samples from Brazil showed similarly high occurrence for type A and B trichothecenes with 31 and 30, respectively (out of 37) having a concentration of >*t*, so both types were found in samples at the same time (which is in stark contrast to South African samples, highlighting the importance of regional assessments). For South America as a whole, the situation was similar, although type A trichothecenes were not as frequently observed; only 40% of samples (*n* = 77) contained both types and more than 70% of samples had an type A trichothecene load of <*t*. South African maize on the other hand, was not contaminated with type A trichothecenes (only 1 sample with a toxin load of 1.6 μg·kg${}^{-1}$ was found to contain type A trichothecenes). T-2 toxin was also absent in a study by Sydenham et al. [84], which were collected Eastern Cape province. On the other hand, 44 out of 53 samples contained type B trichothecenes at concentrations >*t* mirroring results from finished feed.

## 4. Conclusions

The data presented focused on the analysis of 57 mycotoxins and secondary metabolites from regulated, masked and emerging compounds in 1113 samples from three different matrices (i.e., finished feed, maize and maize silage) for the years 2012–2015 obtained as part of a global survey. The single LC-MS/MS analysis method used makes the data well comparable, while providing similar sensitivity for the compounds investigated.

The majority of samples showed low concentrations (i.e., generally below established guidelines for animal feed), however, there were usually a number of samples with high to very high concentrations for all regulated toxins and toxins with guidance levels considered. This highlights the importance of global surveys to maintain concentration levels of commodities below regulatory limits and guidance levels. Observed concentrations are also highly relevant for animal health. For the regulated AFLA, a few individual samples from Africa and Europe showed concentrations exceeding the 20 μg·kg${}^{-1}$ limit. Among the toxins with guidance levels DON, ZEN and to a certain extent FUM remain of global concern with highest concentrations observed, e.g., ZEN and DON concentrations in Central Europe were >*t* in 80% of investigated finished feed samples, i.e., very few uncontaminated samples were observed.

This strongly indicates, that—similar to recently published reports, e.g., by [57]—considerably more than the FAO suggested figure of 25% of global agricultural commodities are contaminated with mycotoxins. In fact >80% of the agricultural commodities could be affected [57,85,86]; in the presented study contamination varied between e.g., 7.1 to 79% for B Trichothecenes and 88% for ZEN. The data of the present study serves as a starting point for a more detailed investigation of contamination rates on a global scale, by also including data and observations from other surveys (see e.g., Table 2). There are multiple issues to consider in an attempt to compare contamination rates. Any increases are in part due to improved analytical methodology and lower limits of detection. Furthermore, the number of samples, their geographical distribution, overall crop yields play a role in the calculation of an updated figure. On the other hand, concentrations at chronic exposure levels having adverse effects on the target population have also changed as new data became available. Other concentration levels, including most of those observed here, e.g., for DON were well below the maximum allowable concentration guidelines.

However, highly sensitive methods provide critical tools to study the adverse impact of low level concentrations of mycotoxins in feed. Results are, therefore, relevant for animal health studies, especially, when synergistic and antagonistic effects of regulated, masked and emerging toxins and secondary metabolites are investigated.

The years 2014 and 2015 showed large increases of yearly median concentrations in Europe. In some regions such as South America, South Africa and the Middle East, FUM played a more significant role with regard to median concentrations than in other regions. Concentrations of OTA play a secondary role with much lower median and maximum concentrations. It has to be noted that even within a fairly small geographic area, e.g., Austria and Italy or Germany and The Netherlands within the Central European region, quite distinct trends and concentration ranges were observed for regulated toxins and toxins with guidance levels. Other parameters such as local meteorological conditions and varieties used need to be considered in order to explain these differences.

The absence of type A trichothecenes from South African samples has to be noted, while the occurrence of type B trichothecenes was quite similar to European samples regarding occurrence and concentrations observed. In general, type A trichothecene concentrations, also exemplified by the sum of T-2 and HT-2 toxin concentrations, were an order of magnitude lower than type B trichothecene concentrations in the very same samples.

Emerging toxins showed high occurrence for enniatins, MON and BEA (e.g., in South Africa), whereas AFLA precursors and ergot alkaloids were much less prevalent with the large majority of samples (up to 90%) showing concentrations of <1 μg·kg${}^{-1}$, if any, i.e., staying below the LOD. In 2014, enniatin concentrations in finished feed were exceptionally high in European samples with an median of 250 μg·kg${}^{-1}$, thus reaching median concentrations in the order of magnitude of DON and ZEN.

While co-occurrence of regulated toxins and toxins with guidance levels with other investigated compounds was frequent and wide-spread for e.g., enniatins, BEA and MON, correlation was limited to relatively few cases, e.g., DON and ZEN with MON in maize samples from South America. An exception was correlation of DON and ZEN with their masked metabolites DON-3-glucoside and ZEN-14-sulfate, which was observed frequently, but not in all cases. AFLA and AFLA precursors were well correlated in samples from Africa.

## 5. Materials and Methods

A total of 1926 samples from 46 countries with concentrations of 380 toxins and metabolites were collected for the years 2012 to 2015. Samples were provided by the Biomin Mycotoxin Survey and analysis was carried out employing a LC–MS/MS multi-mycotoxin analysis method for determination of toxins and metabolites. Of the toxins and metabolites investigated, 162 compounds were detected and quantified. For the presented study data for three different matrices (finished feed, FF; maize, M and maize silage, MSI) and 57 toxins and metabolites were chosen for detailed analysis, resulting in a subset of 1113 samples; see Table 1. These included regulated toxins and compounds with guidance levels, frequently reported in previous surveys, e.g., [57]. Concentrations of masked and emerging toxins and secondary metabolites using a single method of analysis for all mycotoxins and secondary metabolites that were not previously available in a global data set were reported with a focus on co-occurrence with regulated toxins and toxins with guidance levels.

A threshold (*t*) of relevant concentrations was established to be >1.0 μg·kg${}^{-1}$ or the LOD, whichever was higher. For compound groups (e.g., AFLA) the highest LOD in the group was employed [63]. Threshold levels are listed in Table 1. Extracted ion chromatograms of maize samples spiked near the t-value are shown in the supporting information. All plots show the fraction above the threshold unless otherwise indicated.

**Figure 9:**
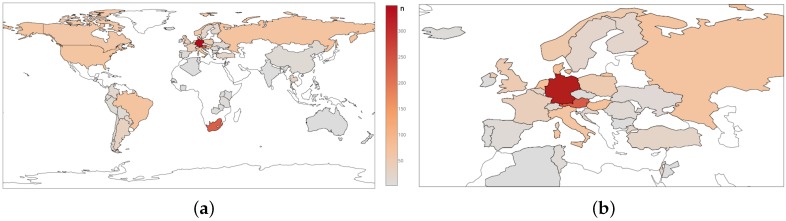
Number of samples n in the investigated data set from a (**a**) Global and (**b**) European perspective.

Figure 9 and Table 5 provide information about sample origin and frequency for each region and country and number of samples analysed in each subset, respectively together with some initial concentration information.

Samples were taken by or under the instruction of trained staff with a protocol for the taking of stratified subsamples, homogenizing and then a minimum 500 g sample being submitted to laboratory. In the laboratory, samples were finely milled and homogenized immediately after reception at the Department of Agrobiotechnology (IFA-Tulln) at the University of Natural Resources and Life Sciences Vienna (BOKU) in Tulln, Austria. Analysis was carried out right after milling and homogenisation. Finished feed (complete feed) for poultry, swine and ruminants were sampled. Composition would be variable both between and within the different feed types.

Samples were analysed using a single “dilute and shoot” LC–MS/MS multi-mycotoxin method previously reported by Malachová et al. [87]. In brief, samples were ground for homogenisation and extracted for 90 min with a mixture of acetonitrile, water and acetic acid (79:20:1, per volume) on a rotary shaker. After centrifugation, the supernatant was transferred into a glass vial, following dilution with solvent (as above, but with a 20:79:1 volume ratio). The extract was injected into a LC–MS/MS system (electrospray ionisation and mass spectrometric detection employing a quadrupole/ion-trap combination mass filter). All samples were analysed using this instrument. Quantification was performed based on external calibration using serial dilutions of a multi-analyte stock solution. Results were corrected for apparent recoveries, which have been determined by spiking experiments using 9 different types of feed (unpublished data). For maize, the respective values obtained in [Malachova2014] were used. Regarding identification and quantitation, the acquisition of two MS/MS transition yields 4.0 identification points according to Commission Decision 2002/657/EC (with the exception of moniliformin and 3-nitropropionic acid, which exhibit only one fragment ion). In addition, the intensity ratios as well as retention times were not to deviate from the respective standards within certain limits, as stated in the same document. However, a fixed ion ratio criteria of 30% and a very strict retention time criteria of ±0.03 min has been applied following a recent suggestion in the field of veterinary drug analysis [88].

The method follows the guidelines established by the Directorate General for Health and Consumer affairs of the European Commission, published in document No 12495/2011 [89]. Apparent recoveries for feed have been determined by spiking experiments using 9 different types of feed (unpublished data). The external verification of the method accuracy by participation in proficiency testing schemes resulted in 104 acceptable, 6 questionable and 2 unsatisfactory results, respectively, for samples of animal feed (results until March 2016 included). While the method has continually evolved over time with more and more species being added, all analytes investigated here, were analysed in the full four year period being considered.

The use of this single unique multi-mycotoxin method provides comparable data for a large number of compounds quantified in samples of global origin. Thus some of the issues raised with previous surveys were overcome that typically employed data from several analytical methods and different analysis protocols (e.g., from several national monitoring agencies, see Table 2).

All concentration data were collected in a single file and sample information such as sampling year and month, country and region of origin and sample matrix were added for subsetting. The complete data set was then imported into Matlab (version R2015b) for subsetting and further statistical analysis. Wilcoxon and Kruskal–Wallis tests of data were completed in R (version 3.3.1) using the “agricolae” package (version 1.2–4). For the latter, a confidence level (CL) of 95% was chosen; if it cold not be attained, a CL of 90% was set instead.

## Figures and Tables

**Table 1:** Summary statistics of investigated concentrations of 57 regulated mycotoxins and mycotoxins with guidance levels, masked and emerging metabolites in 3 matrices (1113 samples from 46 countries). Abbreviations: <LOD—below limit of detection (replaced with zero for the median calculation), *n*—number of samples above threshold *t*, %—percentage of samples above threshold *t*. Indented compound concentrations were summed up as part of a group (group name in italics). A threshold (*t*) of relevant concentrations was established to be >1.0 μg·kg${}^{-1}$ or >LOD, whichever was higher. For sums of compound concentrations, the highest LOD within a group was employed as *t*.

Metabolite (Group)	Median	75th	95th	Maximum	*n* > *t*	% > *t*
	Concentration	Percentile	Percentile	Concentration		
	(μg·kg${}^{-1}$)	(μg·kg${}^{-1}$)	(μg·kg${}^{-1}$)	(μg·kg${}^{-1}$)		
**Regulated toxins and toxins with guidance levels**						
*AFLA, t > 1.5 μg·kg${}^{-1}$*						
Aflatoxin B1	<LOD	<LOD	1.4	1077	49	4.9
Aflatoxin B2	<LOD	<LOD	<LOD	112	14	1.4
Aflatoxin G1	<LOD	<LOD	<LOD	95	19	1.9
Aflatoxin G2	<LOD	<LOD	<LOD	12	8	0.80
ZEN, *t* > 1 μg·kg${}^{-1}$	20	77	596	11,192	884	88
DON, *t* > 1.5 μg·kg${}^{-1}$	193	546	2278	13,488	799	79
*T-2 and HT-2 toxins, t > 10 μg·kg${}^{-1}$*						
T-2 toxin	< LOD	3	22	852	105	10
HT-2 toxin	< LOD	0.0	51	2328	189	19
*FUM, t > 4.0 μg·kg${}^{-1}$*						
Fumonisin B1	42	248	1842	31,784	678	67
Fumonisin B2	14	84	696	12,968	580	58
Fumonisin B3	< LOD	34	284	3345	400	40
Fumonisin B4	< LOD	10	192	4341	284	28
Fumonisin B6	< LOD	< LOD	< LOD	30	1	0.10
OTA, *t* > 1.5 μg·kg${}^{-1}$	< LOD	< LOD	1.0	67	45	4.5
**Masked toxins**						
DON-3-glucoside, *t* > 1 μg·kg${}^{-1}$	12	44	424	3159	701	70
ZEN-14-sulfate, *t* > 2 μg·kg${}^{-1}$	1.3	17	132	4318	471	47
**Emerging toxins**						
BEA, *t* > 1.0 μg·kg${}^{-1}$	8.5	25	114	1610	831	83
MON, *t* > 2.0 μg·kg${}^{-1}$	16	61	236	1367	793	79
*Ergot alkaloids, t > 1.5 μg·kg${}^{-1}$*						
Agroclavine	<LOD	<LOD	<LOD	108	37	3.7
Chanoclavine	<LOD	0.053	0.76	21	19	1.9
Dihydrolysergol	<LOD	<LOD	<LOD	5.2	2	0.20
Elymoclavine	<LOD	<LOD	<LOD	0.24	0	0
Ergine	<LOD	<LOD	<LOD	0.40	0	0
Ergocornine	<LOD	<LOD	1.9	48	56	6
Ergocorninine	<LOD	<LOD	1.8	21	57	5.7
Ergocristine	<LOD	<LOD	13	449	114	11
Ergocristinine	<LOD	<LOD	4.3	118	84	8.3
Ergocryptine	<LOD	<LOD	7.9	65	101	10
Ergocryptinine	<LOD	<LOD	2.0	20	66	6.6
Ergometrine	<LOD	<LOD	12	405	145	14
Ergometrinine	<LOD	<LOD	1.2	53	41	4.1
Ergosine	<LOD	<LOD	6.5	560	102	10
Ergosinine	<LOD	<LOD	1.4	102	49	4.9
Ergotamine	<LOD	<LOD	8.8	334	89	8.8
Ergotaminine	<LOD	<LOD	1.1	65	48	4.8
Festuclavine	<LOD	<LOD	<LOD	22	7	0.70
*Enniatins, t > 1.0 μg·kg${}^{-1}$*						
Enniatin A	0.22	1.5	8.1	92	319	32
Enniatin A1	2.0	10	57	481	596	59
Enniatin B	5.9	29	137	1514	711	71
Enniatin B1	5.4	29	145	1846	693	69
Enniatin B2	<LOD	0.90	4.3	98	233	23
Enniatin B3	<LOD	0.010	0.070	138	30	3.0
*Aflatoxin precursors, t > 4.0 μg·kg${}^{-1}$*						
Norsolorinic acid	<LOD	<LOD	<LOD	24	3	0.30
Averufin	<LOD	<LOD	2.2	139	30	3.0
Averufanin	<LOD	<LOD	<LOD	13	2	0.20
Versicolorin A	<LOD	<LOD	0.12	15	3	0.30
Versicolorin C	<LOD	<LOD	6.1	906	55	5.5
Averantin	<LOD	<LOD	<LOD	9.1	2	0.20
Sterigmatocystin	<LOD	<LOD	1.9	6296	23	2.3
**Trichothecenes**						
*Type A trichothecenes, t > 15 μg·kg${}^{-1}$*						
(incl. T-2, HT-2 toxins)						
Diacetoxyscirpenol	<LOD	<LOD	<LOD	41	2	0.20
15-Monoacetoxyscirpenol	<LOD	<LOD	<LOD	94	7	0.70
Neosolaniol	<LOD	<LOD	2.2	125	35	3.5
T2-Tetraol	<LOD	<LOD	<LOD	290	13	1.3
T2-Triol	<LOD	<LOD	<LOD	93	1	0.10
*Type B trichothecenes, t > 15 μg·kg${}^{-1}$*						
(incl. DON, DON-3-glucoside)						
15-Acetyldeoxynivalenol	<LOD	<LOD	178	2177	128	13
3-Acetyldeoxynivalenol	<LOD	<LOD	24	527	71	7.1
Nivalenol	4.7	18	127	11,232	286	28

**Table 2:** Summary of global survey data for regulated toxins and mycotoxins with guidance levels, masked and emerging toxins. Abbreviations: SBM—soybean meal, DDGS—dried distillers grain with solubles, REG—Toxins and secondary metabolites (regulated or with guidance levels), AFB1—aflatoxin B1, FUS—toxins produced by *Fusarium* spp., NIV—nivalenol, 3-Ac-DON—3-acetyl-DON, DAS—diacetoxyscirpenol, A & B—type A & B trichothecenes, HPLC—high performance liquid chromatography, FLD—fluorescence detector, MS—mass spectrometry, Elisa—enzyme linked immunosorbent assay, TLC—thin layer chromatography, NA—Not available.

Region, Country	Matrix	Year(s)	Toxin	Method	Reference
Global	Feed and ingredients	2004–2013	REG	HPLC, Elisa	[56]
Europe, Asia	Feed and ingredients	2004–2011	REG	HPLC, Elisa	[57]
Global	Feed and ingredients	2004–2012	REG	HPLC, Elisa	[62]
Americas, Europe,	Corn, wheat,	2009–2011	REG	HPLC, Elisa	[58]
Asia	SBM, DDGS				
Middle East, Africa	Feed and ingredients	2009	REG, A&B	HPLC–FLD, LC–MS	[64]
Finland, Sweden,	Cereal grains	1989–2009	REG, NIV,	NA	[63]
Norway, The Netherlands			3-Ac-DON		
Belgium	Oats, pig/poultry feed	2012	FUS	LC–MS/MS	[65]
China	Dairy cow feed	2010	AFLA	HPLC–FLD	[66]
The Netherlands	Maize	2010	REG, FUS	LC–MS/MS	[67]
Pakistan	Poultry feed	2009–2010	AFB1	TLC	[68]
USA	DDGS	2009–2011	REG	HPLC–FLD, TLC	[69]
Portugal	Pig and poultry feed	2009–2010	OTA	HPLC–FLD	[70]
Argentina	Poultry feed	2008–2009	REG, DAS	LC–MS/MS	[71]
South Africa	Compound feeds	2010	REG	LC–MS/MS	[72]
Romania	Cereals	2008–2010	REG	Elisa	[73]
Serbia	Wheat	2007	FUS	LC–MS/MS	[74]
China	Feed and ingredients	2008–2009	REG	LC–MS	[75]

**Table 3:** Number of samples at concentrations >*t* used for the calculation of median plots and statistical analysis. NA—sample number was <1 and no median could be calculated.

Region/Country	Compound	2012	2013	2014	2015
Central Europe					
	AFLA	NA	NA	NA	4
	ZEN	21	20	124	153
	T-2 & HT-2	2	7	57	22
	DON	21	25	123	129
	FUM	6	11	72	94
	OTA	NA	NA	2	5
	Ergots	3	21	84	53
	Enniatins	21	25	128	157
	PreAflas	NA	NA	13	32
	MON	21	19	105	114
	BEA	17	17	98	102
	DON-3-glucoside	20	14	100	121
	ZEN-14-sulfate	2	5	76	92
Austria					
	AFLA	NA	NA	NA	NA
	ZEN	NA	NA	34	24
	T-2 & HT-2	NA	4	24	3
	DON	NA	4	32	22
	FUM	NA	4	24	12
	OTA	NA	NA	NA	NA
Germany					
	AFLA	NA	NA	NA	NA
	ZEN	NA	15	35	33
	T-2 & HT-2	NA	NA	8	9
	DON	NA	15	36	32
	FUM	NA	4	17	14
	OTA	NA	NA	NA	NA
Italy					
	AFLA	NA	NA	NA	NA
	ZEN	2	5	13	34
	T-2 & HT-2	NA	NA	4	4
	DON	2	4	12	32
	FUM	2	4	13	30
	OTA	NA	NA	NA	NA
The Netherlands					
	AFLA	NA	NA	NA	4
	ZEN	NA	2	21	15
	T-2 & HT-2	NA	NA	7	NA
	DON	NA	2	20	13
	FUM	NA	NA	17	12
	OTA	NA	NA	NA	NA

**Table 4:** Summary of co-occurrence and correlation data for investigated samples. For each matrix (finished feed, FF; maize, M; maize silage, MSI) and toxin group (regulated, masked, emerging) the percentage of samples with 3 or more (2 for masked compounds) co-occurring toxins is provided. Correlation between 2 specific species with Pearson coefficients of correlation > 0.5 is listed in the last column. empty cell – not calculated, because of too small subset. (H)T-2 – Sum of T-2 and HT-2 toxins.

Region/Toxins	Co-Occurrence	M (%)	MSI (%)	Correlation
	FF (%)			Compounds (Matrix, $r^{2}$)
**Africa**				
Regulated	100			AFLA & AFLA precursors (FF, 0.93)
				ZEN & Enniatins (FF, 0.54)
				DON & DON-3-glucoside (FF, 0.51)
Masked	92			
Emerging	100			
**South Africa**				
Regulated	90	59		AFLA & AFLA precursors (M, 0.69)
				AFLA & BEA (M, 0.77)
				DON & DON-3-glucoside (M, 0.76)
				ZEN & ZEN-14-sulfate (M, 0.65)
Masked	50	28		
Emerging	90	36		
**Central Europe**				
Regulated	73	56	54	DON & DON-3-glucoside (FF, 0.57)
				DON & DON-3-glucoside (M, 0.80)
				ZEN & ZEN-14-sulfate (M, 0.56)
				DON & DON-3-glucoside (MSI, 0.77)
				ZEN & ZEN-14-sulfate (MSI, 0.79)
Masked	57	72	44	
Emerging	93	83	83	
**Eastern Europe**				
Regulated	74			(H)T-2 & DON-3-glucoside (FF, 0.50)
				DON & DON-3-glucoside (FF, 0.81)
				ZEN & ZEN-14-sulfate (FF, 0.76)
				(H)T-2 & BEA (FF, 0.57)
Masked	22			
Emerging	91			
**Northern Europe**				
Regulated	45			DON & DON-3-glucoside (FF, 0.58)
				ZEN & ZEN-14-sulfate (FF, 0.65)
Masked	47			
Emerging	82			
**Southern Europe**				
Regulated	89			DON & DON-3-glucoside (FF, 0.63)
Masked	73			
Emerging	96			
**Middle East**				
Regulated	93			DON & DON-3-glucoside (FF, 0.50)
				FUM & MON (FF, 0.51)
Masked	91			
Emerging	95			
**North America**				
Regulated	88	63		DON & DON-3-glucoside (FF, 0.50)
				ZEN & ZEN-14-sulfate (FF, 0.81)
Masked	52	40		
Emerging	100	77		
**South America**				
Regulated		54		AFLA & AFLA precursors (M, 0.98)
				DON & DON-3-glucoside (M, 0.76)
				ZEN & ZEN-14-sulfate (M, 0.93)
				DON & MON (M, 0.56)
				ZEN & MON (M, 0.64)
Masked		21		
Emerging		71		

**Table 5:** (a) Maximum (Max.) and average (Av.) number (no.) of compounds found in samples and defined subsets; (b) Number of samples in region and country subsets. Unless explicitly noted, only subsets with 40 or more samples (in bold in the table) were employed for detailed analysis in order to ensure representativity. Statistical data available in Table 1; (c) Countries assigned to regions used for analysis of data. Data for regions in italics were not reported due to a too small sample size (with the exception of Africa).

**(a)**		**Number of Metabolites**	
**All 1926 Samples,**		**All Matrices**	
**380 compounds measured,**			
**162 quantified**			
Max. no. of compounds per sample		68	
Compounds in samples, conc. >1 μg·kg${}^{-1}$		59	
Av. no. of compounds in all samples		28	
Av. no. of compounds, conc. >1 μg·kg${}^{-1}$		24	
		**Number of metabolites**	
**Subset 1113 samples,**	**Finished feed**	**Maize**	**Maize silage**
**57 metabolites quantified**			
Max. no. of compounds per sample	35	29	28
Compounds in samples, conc. >1 μg·kg${}^{-1}$	31	26	20
Av. no. of compounds in all samples	16	12	11
Av. no. of compounds, conc. >1 μg·kg${}^{-1}$	13	10	9
**(b)**		**Number of Samples**	
	**Finished Feed**	**Maize**	**Maize Silage**
**Region subsets**			
All samples	708	267	138
Africa	24	7	1
South Africa	**74**	**53**	28
Central Europe	**335**	**76**	**78**
Eastern Europe	**45**	9	1
Northern Europe	**68**	4	12
Southern Europe	**90**	11	2
Middle East	23	0	0
North America	27	30	15
South America	22	**77**	1
**Country subsets**			
Austria	**64**	18	26
Germany	**89**	32	38
Hungary	**67**	2	3
Italy	**53**	2	2
The Netherlands	**40**	7	9
**(c) Countries in region (total of 46)**			
**Africa**: Algeria, Ivory Coast, Kenya, Senegal, Tunisia, Tanzania, Uganda, Zambia			
**South Africa**: South Africa			
**Central Europe**: Austria, Belgium, Czech Republic, France, Germany, Hungary, The Netherlands, Poland,			
Romania, Switzerland			
**Eastern Europe**: Bulgaria, Russia, Ukraine			
**Northern Europe**: Denmark, Finland, Iceland, Ireland, Norway, Sweden, United Kingdom			
**Southern Europe**: Croatia, Italy, Spain, Portugal, Turkey			
**Middle East**: Israel, Jordan			
**North America**: United States, Canada			
**South America**: Argentina, Brazil, Bolivia, Chile, Colombia, Ecuador, Paraguay, Peru			

## Abbreviations

The following abbreviations are used in this manuscript:

3-Ac-DON: 3-acetyl-deoxynivalenol
A & B: type A & B trichothecenes
AF: aflatoxin
AFB1: aflatoxin B1
AFLA: aflatoxins
BEA: beauvericin
CL: confidence level
DAS: diacetoxyscirpenol
DDGS: dried distillers grain with solubles
DON: deoxynivalenol
Elisa: enzyme linked immunosorbent assay
EU: European Union
FAO: Food and Agriculture Organization of the United Nations
FF: finished feed
FLD: fluorescence detector
FUM: fumonisins
FUS: toxins produced by *Fusarium* spp.
HPLC: high performance liquid chromatography
LC–MS/MS: liquid chromatography tandem mass spectrometry
LOD: limit of detection
M: maize
MON: moniliformine
MS: mass spectrometry
MSI: maize silage
NIV: nivalenol
OTA: ochratoxin A
REG: toxins and secondary metabolites (regulated or with guidance levels)
SBM: soybean meal
TLC: thin layer chromatography
ZEN: zearalenone
